# Upscale of recombinant α-L-rhamnosidase production by *Pichia pastoris* Mut^S^ strain

**DOI:** 10.3389/fmicb.2015.01140

**Published:** 2015-10-19

**Authors:** Kristína Markošová, Lenka Weignerová, Michal Rosenberg, Vladimír Křen, Martin Rebroš

**Affiliations:** ^1^Department of Biochemical Technology, Slovak University of TechnologyBratislava, Slovakia; ^2^Laboratory of Biotransformation, Institute of Microbiology, Academy of Sciences of the Czech RepublicPrague, Czech Republic

**Keywords:** *Pichia pastoris*, α-L-rhamnosidase, recombinant enzyme, fermentation, upscale

## Abstract

*Pichia pastoris* is currently one of the most preferred microorganisms for recombinant enzyme production due to its efficient expression system. The advantages include the production of high amounts of recombinant proteins containing the appropriate posttranslational modifications and easy cultivation conditions. α-L-Rhamnosidase is a biotechnologically important enzyme in food and pharmaceutical industry, used for example in debittering of citrus fruit juices, rhamnose pruning from naringin, or enhancement of wine aromas, creating a demand for the production of an active and stable enzyme. The production of recombinant α-L-rhamnosidase cloned in the Mut^S^ strain of *P. pastoris* KM71H was optimized. The encoding gene is located under the control of the AOX promoter, which is induced by methanol whose concentration is instrumental for these strain types. Fermentation was upscaled in bioreactors employing various media and several methanol-feeding strategies. It was found that fed batch with BSM media was more effective compared to BMMH (Buffered Methanol-complex Medium) media due to lower cost and improved biomass formation. In BSM (Basal Salt Medium) medium, the dry cell weight reached approximately 60 g/L, while in BMMH it was only 8.3 g/L, without additional glycerol, which positively influenced the amount of enzyme produced. New methanol feeding strategy, based on the level of dissolved oxygen was developed in this study. This protocol that is entirely independent on methanol monitoring was up scaled to a 19.5-L fermenter with 10-L working volume with the productivity of 13.34 mg_prot_/L/h and specific activity of α-L-rhamnosidase of 82 U/mg. The simplified fermentation protocol was developed for easy and effective fermentation of *P. pastoris* Mut^S^ based on dissolved oxygen monitoring in the induction phase of an enzyme production.

## Introduction

*Pichia pastoris* is a methylotrophic yeast used as an expression host for the production of recombinant proteins. The gene encoding for the protein is located under the control of the alcohol oxidase (AOX) promoter, which is induced by methanol and repressed by glycerol. Methanol, a source of carbon and energy, is utilized in peroxisomes by two enzymes AOX and dihydroxyacetone synthase. Methanol is transported and oxidized into formaldehyde with the parallel production of hydrogen peroxide (Cereghino and Cregg, 2000). Since formaldehyde is toxic and higher methanol concentrations retard the growth and biomass yield (Khatri and Hoffmann, 2006) it is important to set the methanol induction properly. *P. pastoris* expresses recombinant proteins both intracellularly and extracellularly, which simplifies protein isolation from the production media. In addition, the concentration of produced proteins is quite high and the posttranslational modifications are superior to those in prokaryotic expression systems (Cereghino et al., 2002).

The cultivation of *Pichia* is divided into two steps. To obtain a high cell biomass density, the yeast is first grown in a rich medium with a high concentration of glycerol. After glycerol utilization, methanol feeding starts in a fed-batch mode of operation. Various methanol-feeding strategies have been reported for *Pichia* fed-batch cultivation, such as methanol adaptation steps (Dietzsch et al., 2011a). A mixed-feed strategy has also been reported (Zalai et al., 2012), where both glycerol and methanol are added at the beginning of cultivation. Intensive studies have also focused on substrate uptake rates to improve the metabolism of *P. pastoris* by glucose- and methanol-feeding strategies (Dietzsch et al., 2011a).

In this study, the recombinant extracellular α-L-rhamnosidase (EC 3.2.1.40), originating from *Aspergillus terreus*, was expressed in *P. pastoris*. No upscale of this enzyme by *P. pastoris* was reported so far. Only the low scale expressions in recombinant strains of *E. coli* (Kaur et al., 2010; Zhang et al., 2015) or in various native producers (Yanai and Sato, 2000) were demonstrated. The strain *P. pastoris* KM71H was transformed by a linearized expression vector containing the α-L-rhamnosidase gene. The effective overexpression was optimized (recombinant α-L-rhamnosidase was produced as an extracellular protein at a 3× higher activity and in half the time compared to the natural producer; Gerstorferová et al., 2012). Compared to the native enzyme, the recombinant one exhibited a higher activity under alkaline conditions (up to pH 11.5), which is very important for the solubility of the flavonoids used as substrates. This recombinant enzyme has an extremely broad and rather flat pH profile (1.5–11.5), which is a major advantage in biotechnological applications. Moreover, it is stable at temperatures up to 80°C. Recombinant α-L-rhamnosidase has been expressed in very high purity and void of undesired β-D-glucosidase activity (Gerstorferová et al., 2012). This enzyme is used for the production of L-rhamnose from rutin (Perez et al., 2003). Some rhamnosides are important bioactive compounds, including cytotoxic saponins (Yu et al., 2002), antifungal plant glycoalkaloids (Oda et al., 2002), and bacterial virulence factors (Deng et al., 2000). Clear correlations between the presence of specific sugar residues and the biological activity of these molecules have been demonstrated in a plethora of examples (Křen and Martínková, 2001).

Recently, α-L-rhamnose and/or α-L-rhamnosides were found to interact with specific receptors on keratinocytes, which play an important role in cell and (skin) tissue aging (Faury et al., 2011). This finding triggered vigorous research in the cosmetic application of rhamnosides, and indeed the application of rhamnose and rhamnosides was patented (Laboureau et al., 2010) and these preparations reached the market. Subsequently, it was found that rhamnose itself does not penetrate into the skin, but when conjugated (e.g., in the form of pentyl α-L-rhamnoside) its concentration in the stratum corneum and epidermis is doubled (Duplan et al., 2009). This clearly demonstrates the importance of the preparation of rhamnosides, especially by enzymatic methods, which do not involve any harmful and irritant chemicals, thereby making these products applicable in the cosmetics and food industries.

This work focused on upscaling of recombinant α-L-rhamnosidase production using the Mut^S^ *P. pastoris* KM71H strain by simplified methanol feeding protocol. Since α-L-rhamnosidase is an important enzyme in the food and pharmaceutical industry there is well-grounded demand for its up scale production. The optimization of growth, the production media and different methanol feeding strategies were studied. The results are divided into three parts. In the first one, the flask experiments were performed as a starting point according to previous results (Gerstorferová et al., 2012). The second part focus on the fermentations with fed batch methanol feeding and essential off-line methanol measurements. In the third part the simplified fermentation protocol was developed. It linked the methanol feeding with the level of dissolved oxygen without the necessity of off-line methanol concentration monitoring.

## Materials and Methods

### Microorganism

The recombinant *P. pastoris* strain KM71H (Invitrogen, USA) transformed with α-L-rhamnosidase from *Aspergillus terreus* CCF 3059 has been described elsewhere (Gerstorferová et al., 2012).

### Media

The production strain of *P. pastoris* Mut^S^ was cryopreserved at -80°C in 15% (v/v) glycerol and cultivated on YPD [Yeast Extract Peptone Dextrose Medium: 1% (w/v) yeast extract, 2% (w/v) peptone, 2% (w/v) glucose, and 2% (w/v) agar] plates. The inoculum both for the flasks and for the fermenter experiments was grown in BMGY medium [Buffered Glycerol-complex Medium: 1% (w/v) yeast extract, 2% (w/v) peptone, 100 mM potassium phosphate, pH 6.0, 1.34% (w/v) YNB (Yeast Nitrogen Base, Invitrogen, USA), 4 × 10^-5^ % (w/v) biotin, 1% (v/v) glycerol]. The production of the enzyme was performed in the production medium BMMH [Buffered Methanol-complex Medium, the same as BMGY but 0.5% (v/v) methanol is added instead of 1% (v/v) glycerol]. Fed-batch fermentations were carried out in BSM (Basal Salt Medium: 26.7 mL/L 85% H_3_PO_4_, 1.17 g/L CaSO_4_⋅ 2 H_2_O, 18.2 g/L K_2_SO_4_, 14.9 g/L MgSO_4_⋅ 7 H_2_O, 4.13 g/L KOH, and 40 g/L glycerol) and supplemented with 4.35 mL of PTM_1_ (trace salts solution: 6 g/L CuSO_4_⋅ 5 H_2_O, 0.08 g/L NaI, 3 g/L MnSO_4_⋅ H_2_O, 0.2 g/L Na_2_MoO_4_⋅ 2 H_2_O, 0.02 g/L H_3_BO_3_, 0.5 g/L CoCl_2_, 20 g/L ZnCl_2_, 65 g/L FeSO_4_⋅ 7 H_2_O, 0.2 g/L biotin, 9.2 g/L H_2_SO_4_) per liter of BSM medium. Methanol added in fed-batch experiments was also supplemented with PTM_1_ (1.2 mL/L pure methanol).

### Preculture

A final volume of 100 mL of BMGY in 500-mL flasks was inoculated with a single colony from YPD plates and cultivated for 24 h in a rotary shaker (220 rpm, 28°C).

### Batch Cultivations with Methanol Pulses

Batch fermentations were carried out in 500-mL flasks with 100 mL of BMMH media and in 1.3-L Brunswick BioFlo^®^ 115 fermenters (Eppendorf, Hamburg, Germany) with 0.5 L of BMMH media. In the flask experiments, 100 mL of biomass from the inoculum was centrifuged (10 min, 4°C, 7,197 g) and resuspended in 100 mL of BMMH. Cultivation was carried out on a rotary shaker at 220 rpm and 28°C. Once every 24 h, 1 mL of pure methanol per 1 L of medium was added. In laboratory fermenters, the conditions were as follows: 20% v/v inoculum (prepared as described above), DO 40% with cascade agitation 50–1000 rpm, 28°C, pH 6.0 maintained with an ammonia solution. As an antifoaming agent, 200 μL of Struktol J650 (Schill + Seilacher “Struktol” GmbH, Hamburg, Germany) per 1 L of medium was used. Methanol (1 mL/L) was added in the same way as in the flask experiments, including adding 1 mL of pure methanol per 1 L of medium once every 24 h.

### Fed-batch Cultivation and Methanol-Feeding Strategies

Fed-batch cultivations were carried out in 3-L laboratory fermenters (Brunswick BioFlo^®^ 115) with 1.5 L of BSM medium. Methanol feeding started after the complete utilization of glycerol. Methanol-feeding strategies included: pulsed addition in 24-h intervals (or when the methanol was depleted); fed-batch feeding according to the actual (off-line) methanol concentration; fed-batch feeding according to the actual level of dissolved oxygen; and continuous feeding. In different experiments, different fermentation conditions were set, which we subsequently describe in detail.

### Upscale Cultivation

Upscale fermentations were performed in a 19.5-L laboratory fermenter (New Brunswick Scientific BioFlo^®^ 510, Eppendorf, Hamburg, Germany) with 10 L of BSM medium sterilized *in situ*. Conditions were set as follows: 5% v/v inoculum (prepared as described above), DO 20% with cascade agitation 100–400 rpm, 30°C, pH 5.0 maintained with an ammonia solution, overpressure 0.5 bar and 1 vvm of aeration. As an antifoaming agent, 200 μL of Struktol J650 was used for each 1 L of medium. Methanol fed-batch feeding was controlled according to the actual level of dissolved oxygen by an automated program. After two methanol pulses (3 g/L) the program was set to following parameters: if the DO was >30%, the methanol flow is set to 69 mL/h, and if the DO is >40%, the feeding was stopped.

### Enzyme Activity Assay

The activity of expressed α-L-rhamnosidase was measured as described before (Gerstorferová et al., 2012). The substrate *p*-nitrophenyl α-L-rhamnopyranoside (*p*NP-α-L-Rha; Sigma–Aldrich, USA) was used. One unit of enzymatic activity was defined as the amount of enzyme releasing 1 μmol of *p*-nitrophenol (*p*-NP-OH) per minute in 50 mM citrate-phosphate buffer at pH 6.0 and 35°C. After incubation of the enzyme with the substrate at 35°C, and agitation at 900 rpm for 10 min, the reaction was stopped with the addition of 1 mL of 0.1 M Na_2_CO_3_ and the amount of released *p*-nitrophenol was measured spectrophotometrically (UV-VIS Spectrophotometer Agilent 8453, Agilent Technologies, Germany) at 420 nm. All measurements were performed in triplicate.

### Analysis

Biomass growth was measured spectrophotometrically (BioSpectrophotometer, Eppendorf, Hamburg, Germany) at 600 nm. Dry cell weight (DCW) was calculated from the correlation *y* (DCW in g/L) = 0.2001 × (OD_600_) + 0,1075 obtained experimentally for this strain. The level of expressed proteins was analyzed by 10% sodium dodecyl sulfate polyacrylamide gel electrophoresis (SDS-PAGE) followed by bromophenol blue staining and also by the Bradford method (Bradford, 1976). After fermentation, the culture was centrifuged and the supernatant was filtered through a non-pyrogenic sterile filter (Filtropur S 0.2 μm, Sarstedt).

The amounts of glycerol and methanol were measured by HPLC with an Agilent Technologies 1220 Infinity LC apparatus with an Agilent Technologies 1260 Infinity RI detector (Agilent Technologies, Germany). The column was a WATREX Polymer IEX H form 8 μm, 250 × 8 mm and the guard column was a WATREX Polymer IEX H form 8 μm, 40 × 8 mm, at a flow rate of 0.8 mL/min of 9 mM sulfuric acid at 45°C.

## Results And Discussion

### Flask Experiments

Flask experiments were carried out to verify the results from *Pichia* cultivation according to Gerstorferová et al. (2012). After a 24-h cultivation of yeast on glycerol medium (BMGY), the culture reached 2.51 g/L of DCW. The biomass was centrifuged, resuspended in 100 mL of BMMH medium, and 1 mL of methanol was added every 24 h. After 3 days of induction, the maximum specific activity reached 5 U/mL, and it did not change over the next 2 days (**Table 1**). This was lower than the previously published maximum achieved activity of 9 U/mL (Gerstorferová et al., 2012). The maximum DCW was 8.31 g/L.

**Table 1:** Comparison of growth and activity in BMMH (Buffered Methanol-complex Medium) with various initial glycerol concentrations.

Inoculum (% v/v)	Initial glycerol (g/L)	DCW (g/L) (24 h)	Maximum DCW (g/L)	μ_max_ (h^-1^)	Final activity (U/ml)	Fermentation time (*h*)	Methanol feeding intervals (hours)
**Flask experiment**							
100	0	4.31	8.31	0.02	5	120	24
**Fermenter experiments**							
20	0	1.54	3.11	0.02	2.3	115	24
20	12.6	13.71	13.71	0.18	8.2	115	24
10	12.6	11.11	11.71	0.22	5.7	185	12
5	12.6	12.31	21.72	0.25	24	185	4

### Batch Experiments in BMMH Medium

**FIGURE 1:**
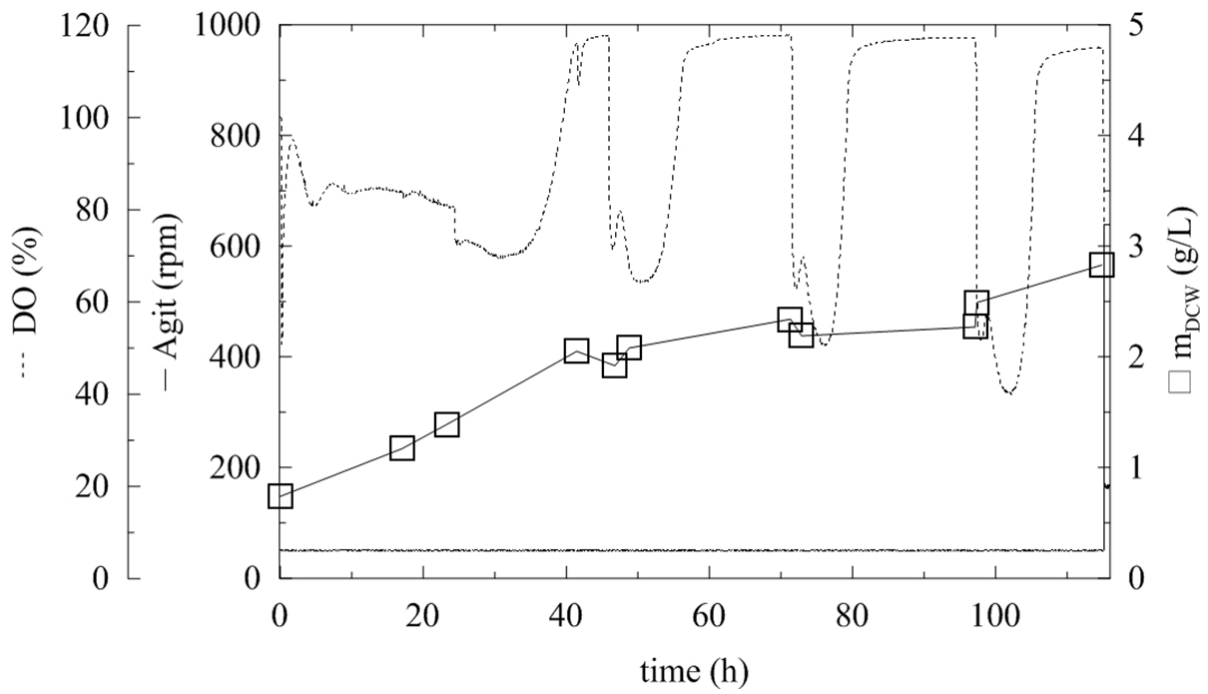
**Fermentation of *Pichia pastoris* expressing α-L-rhamnosidase with methanol pulses every 24 h.** Conditions: 0.5 L BMMH, 28°C, pH 6 (ammonia solution), DO = 40% by agitation cascade 50–1,000 rpm, 20% inoculum.

The production and activity of an enzyme are influenced by various fermentation parameters, which need to be optimized separately for each expressed protein. The previous flask experiments were therefore upscaled to 0.5-L working volume fermenters, where cells could grow under controlled conditions. For these experiments, a 20% (v/v) inoculum was used, and the DO was maintained at 40% saturation by an agitation cascade. Even after 120 h of fermentation, the DCW reached its maximum of 3.11 g/L (**Figure 1**; **Table 1**). This was due to using methanol as the sole carbon source, since *P. pastoris* KM71H is a Mut^S^ strain (methanol utilization slow) (Zhang et al., 2003) and has a significantly lower biomass growth rate and yield on this substrate compared to glycerol as a carbon source. The maximum activity was half of the activity achieved in flasks (2.3 U/mL) after 115 h. This resulted from a lower cell density in the methanol production medium, since methanol was the sole substrate for biomass growth and also for the enzyme production phase (**Table 1**).

To increase biomass growth, the initial addition of glycerol (12.6 g/L) to the production BMMH medium and various inocula (5, 10, and 20 % v/v) was tested (**Table 1**). It was proven that the addition of glycerol had a considerable effect on biomass growth. With the same methanol-feeding strategy as in the flasks (pulses every 24 h, 1 mL of pure methanol per 1 L of media) the maximum activity at the end of cultivation, in the 115th hour, was 8.2 U/mL. As described above after methanol addition, the DO curve sharply decreased, but increased again after 5 h upon methanol depletion. Until the next methanol pulse, *Pichia* was “starving” suggesting that these methanol pulses were insufficient for the induction of α-L-rhamnosidase. Therefore, a second methanol-feeding strategy was applied, with pulse methanol addition when the oxygen levels began to increase (approximately every 4 h). After 185 h of fermentation, the activity reached 24 U/mL, which was three times higher than in previous experiments. The amount of inoculum used slightly affected the specific growth rate (**Table 1**) but had no meaningful influence on the final biomass concentration in the 24th hour in glycerol. Therefore, in all further experiments 5% v/v inocula were used. The specific growth rate was approximately 10 times less in methanol as the sole carbon source (**Table 1**), which has been previously reported (Zhang et al., 2003). The methanol-feeding strategy had a significant influence on the final biomass concentration and also on the final enzymatic activity (**Table 1**).

### Fed-batch Methanol-feeding Strategy

Since there were no significant changes in the activity of the produced enzyme, the fermentation medium BMMH was changed to a defined BSM (Dietzsch et al., 2011a). The initial concentration of glycerol (20, 40, 60 g/L) as the carbon source was the first parameter tested. As expected, the initial glycerol concentration affected several fermentation parameters (**Table 2**). A lower initial glycerol concentration led to a higher maximum specific growth rate (**Table 2**). This corresponds with the published data on glycerol as an initial substrate for *Pichia* growth (Jahic et al., 2003; Zhang et al., 2003). When *Pichia* grows in a methanol-containing medium, the specific growth rate significantly decreases to 0.08 h^-1^, as previously reported for tests of *P. pastoris* GS115 (Mut^+^) in BSM media (at a methanol concentration of 3.65 g/L) (Zhang et al., 2000). The maximum DCW also increased with an increase in the glycerol concentration (**Table 2**).

**Table 2:** Comparison of growth in BSM (Basal Salt Medium) with various initial glycerol concentrations.

Initial glycerol (g/L)	Glycerol utilized		Max. DCW (g/L)	μ_max_ (h^-1^)
	Time (h)	Reached DCW (g/L)		
20	18	19.12	38.13	0.27
40	24	33.52	67.74	0.23
60	25	38.13	71.34	0.18

**FIGURE 2:**
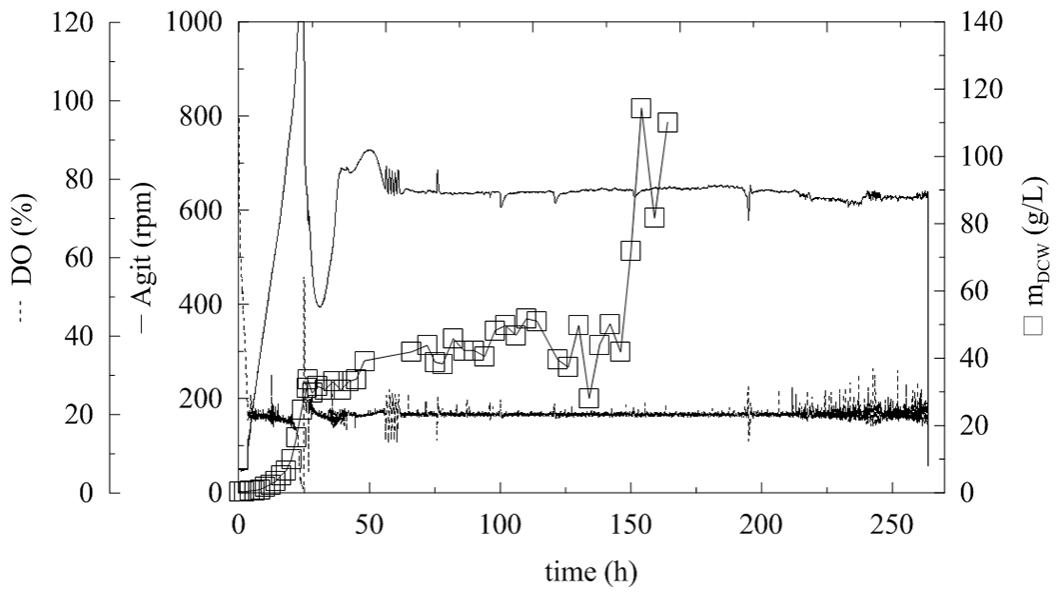
**Fermentation of *P. pastoris* expressing α-L-rhamnosidase with continual methanol feeding.** Conditions: 1.5 L BSM (Basal Salt Medium), 30°C, pH 5 (ammonia solution), DO = 20% by agitation cascade 50–1,000 rpm, 5% inoculum.

As described above, higher protein expression and final enzyme activities correlated with higher OD values. Maximum dry cell weights are lower than with applying fed-batch glycerol phase (120 g/L, Xie et al., 2003), but comparable with other Mut^S^ strains, when the glucose as substrate was used (70 g/L, Dietzsch et al., 2011b). It has been shown that the addition of PTM_1_ has a significant influence on the production of recombinant enzymes by *P. pastoris* KM71H in fed-batch processes (Wanderley et al., 2013), when the production of recombinant frutalin was tested. The temperature was set to 30°C, DO to 20 %, the pH was maintained at 5.0 with an ammonia solution and the initial glycerol concentration used was 40 g/L (**Figure 2**).

**FIGURE 3:**
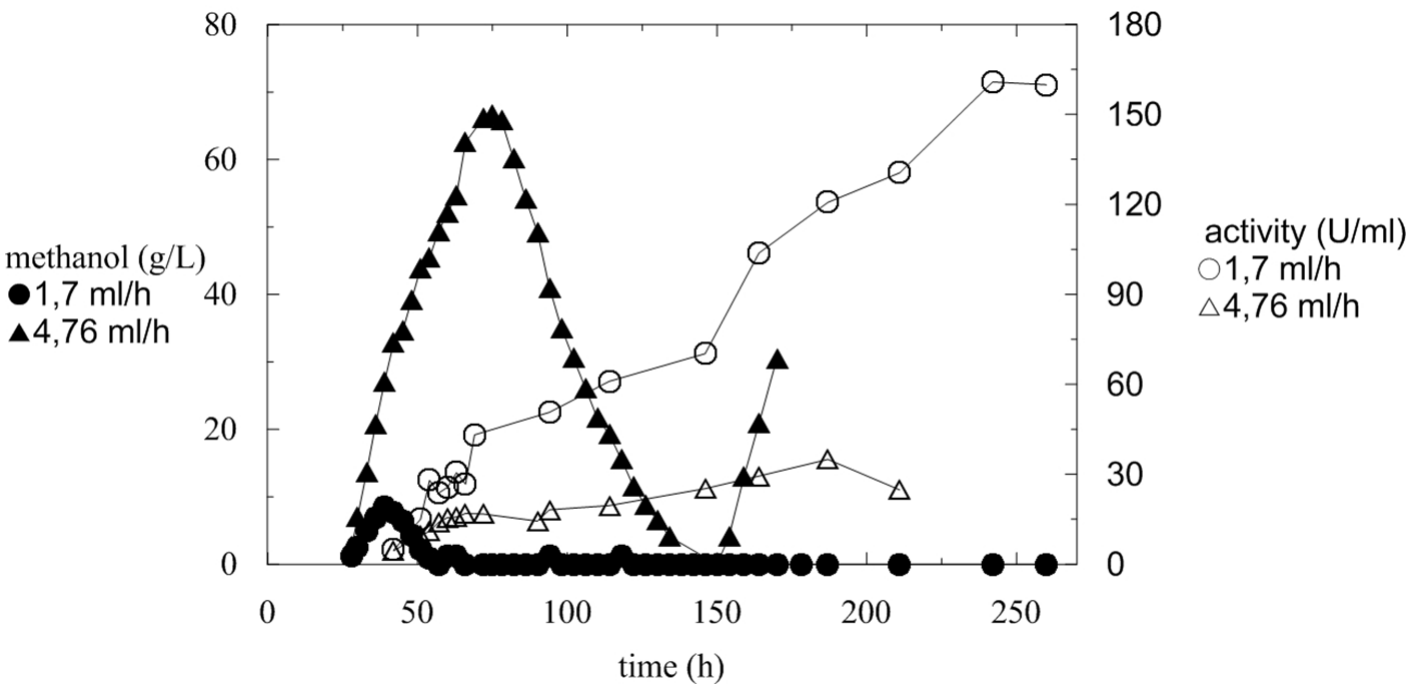
**Comparison of increase in activity during fermentation with variable methanol feeding flow and monitoring of methanol concentration**.

After the complete depletion of glycerol (40 g/L; 24 h of fermentation), the DO tended to increase. Therefore, the cascade agitation decreased from its maximum equal to 1000 rpm. Methanol feeding began at this point. Fed-batch methanol feeding started with a continuous flow at two different average feeding rates of 1.7 and 4.76 mL/h. The DCW after 24 h of fermentation was 20.32 and 24.52 g/L, respectively, which was double that in BMMH medium containing glycerol. Methanol feeding at 1.7 mL/h resulted in an increase in the methanol concentration to 9 g/L (**Figure 3**). After 20 h the methanol was completely utilized as a new substrate. When a methanol flow of 4.76 mL/h was used, the concentration of methanol in the production medium started to increase exponentially. This was caused by the slow metabolism of the Mut^S^ strain (a strain that slowly utilizes methanol), which was not able to utilize methanol at such a high rate, and resulted in an increase in the methanol concentration in the medium. As described previously, the concentration of methanol should not exceed 3.65 g/L, because an excess of methanol and oxygen leads to the accumulation of formaldehyde and hydrogen peroxide to toxic levels (Zhang et al., 2003; Khatri and Hoffmann, 2006). Therefore, when the methanol level reached 66 g/L, feeding was interrupted until the methanol concentration decreased to 0 g/L, and then the feeding was reactivated. This again led to an increase in methanol concentration in the fermentation medium, because *Pichia* still had not adapted to such a high methanol feed rate. Furthermore, when the methanol concentration decreased to below 5 g/L, the biomass concentration significantly increased (**Figure 2**).

At a higher methanol flow rate (4.76 mL/h) (**Figure 3**; **Table 3**, Batch A), the amount of accumulated methanol inhibited the expression of active α-L-rhamnosidase, which reached a maximum of 34 U/mL, 35 U/mg_prot_ and the protein productivity of 3.79 mg/L/h. In comparison with a flow rate of 1.7 mL/h, methanol was utilized continuously and completely and the activity increased almost linearly. The maximum activity was 160 U/mL after 240 h (**Figure 3**; **Table 3**, Batch B).

**Table 3:** Summary of *Pichia pastoris* fermentation with various methanol-feeding strategies.

	c_s_ (g/L)	Ad	FR_met_ (ml/h)	c_met_ (g/L)	F_met_ (ml)	EA_200_ (U/ml)	*t* (h)	EA_max_ (U/ml)
A	40	–	4.76	65	450	24.8	211	34
B	40	–	1.7	7.7	400	120.6	260	160
C	40	+	2.46	4.3	800	109.0	501	322
D	40	+	*p*	16.2	496	136.6	323	200
E	20	+	4.6	74.2	560	57.4	262	62
F	60	+	0.57	n.a.	370	125.4	262	166
G	40	+	–	17.4	789	162.6	260	214
H	40	+	–	3	2220	164.0^∗^	196	164

*c_*s*_ – initial glycerol; Ad – two pulse methanol adaptation steps; c_*met*_ – maximum methanol concentration in fermentation broth; FR_*met*_ – methanol feeding rate in continuous mode; F_*met*_ – total amount of fed methanol; EA_*200*_ – enzyme activity after 200 h of fermentation; t – total duration of fermentation; EA_*max*_ – maximum enzyme activity reached; n.a. – not available; p – fed-batch pulses; ^∗^ – in the 196th hour.*

**FIGURE 4:**
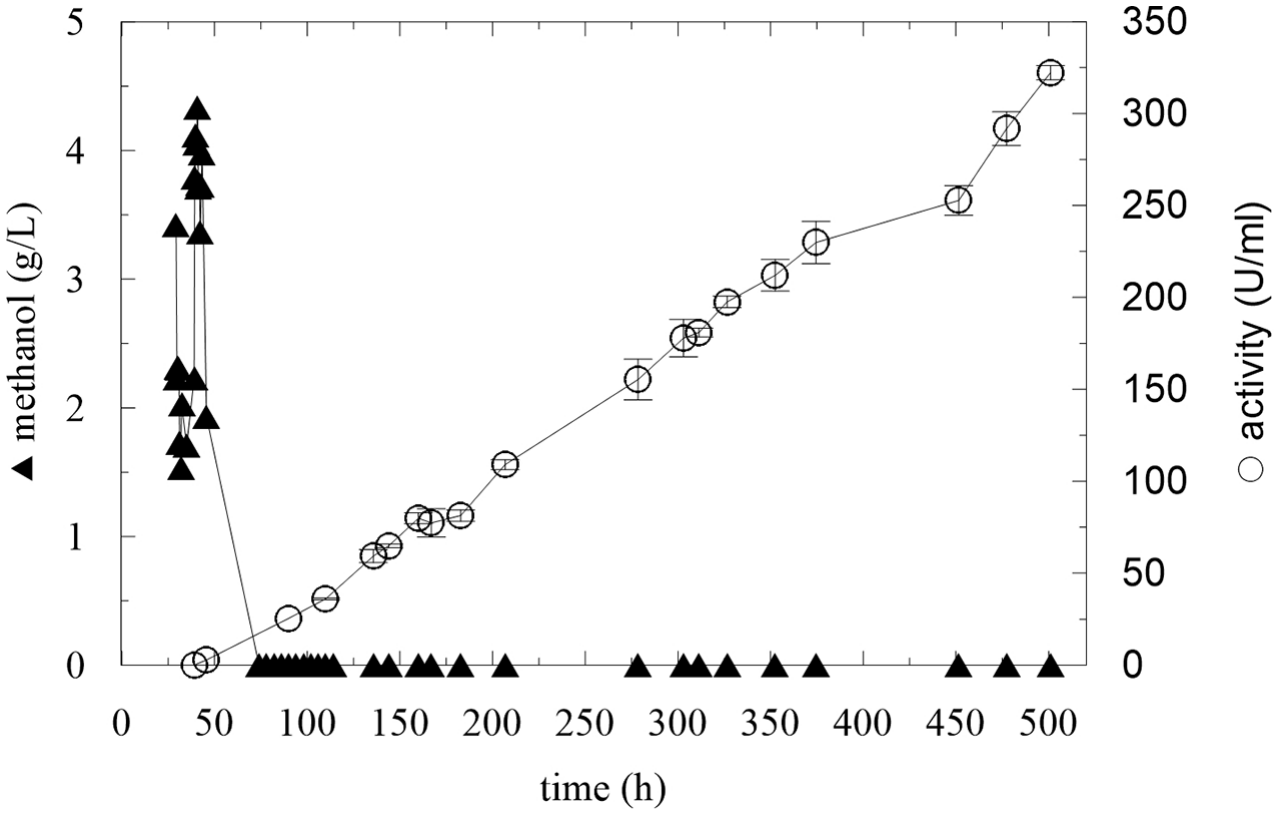
**Pulse adaptation of culture to methanol and continuous methanol feeding**.

These results show that the strain needs to adapt to methanol prior to initiating the methanol feeding. In the next experiment, an adaptation step was added, using two methanol pulses before continuous feeding, according to previously published findings (Dietzsch et al., 2011a). After all the glycerol was utilized, the first methanol pulse (final methanol concentration 3.4 g/L) was applied. After 10 h, the second pulse was applied and after the next methanol utilization, continuous feeding was initiated at a flow rate of 2.46 mL/h with a methanol stock concentration of 50% v/v (**Table 3**, Batch C). **Figure 4** demonstrates that the culture was sufficiently adapted to methanol, because the enzyme activity increased linearly. Since the methanol concentration in the medium remained at 0 g/L, at 206 h, the stock methanol concentration was changed to 100% v/v. After this point, the activity still increased with a methanol concentration of 0 g/L, which means that the culture was perfectly adapted and it was able to utilize higher amounts of methanol.

**FIGURE 5:**
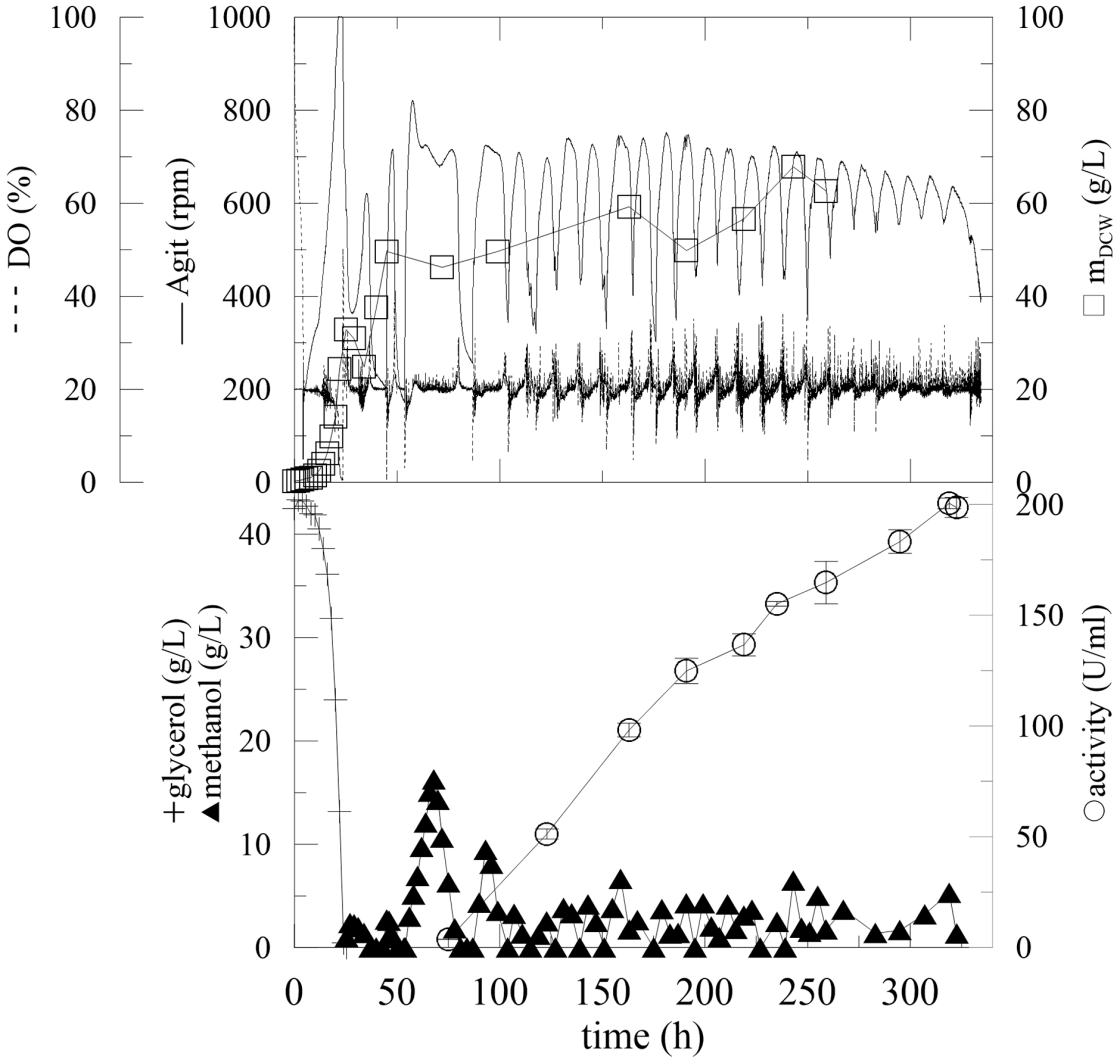
**Fermentation of *P. pastoris* expressing α-L-rhamnosidase with fed-batch methanol feeding linked with oﬄine methanol monitoring.** Conditions: 1.5 L BSM, 30°C, pH 5 (ammonia solution), DO = 20% by agitation cascade 50–1,000 rpm, 5% inoculum. Monitoring of concentration of methanol present in fermentation cultures with fed-batch methanol feeding linked with oﬄine methanol monitoring.

To avoid the problems of excess methanol in the production media, another feeding strategy was tested. It was based on the off-line actual methanol measurements and changes in the methanol feed rate were set up according to the current methanol concentration. After the glycerol was depleted and two starvation steps were included, continuous methanol feeding was initiated (**Figure 5**; **Table 3**, Batch D).

Maintaining the methanol concentration at a low level (under 5 g/L), inhibited the biomass growth. Compared to previous experiments, however, this strategy had no significant influence on enzyme activity, because the activity at 350 h was the same in both experiments (**Figure 5**). Another fermentation with different concentrations of glycerol and different methanol-feeding rates were performed, but no significant improvement of the process was observed (**Table 3**, Batch E, F). The specific productivities of total extracellular proteins were in range of 6–12 mg/L/h. According to SDS-PAGE, dominantly α-L-rhamnosidase and another one minor protein were observed to be produced (data not shown).

#### Methanol Induction Based on DO Consumption

**FIGURE 6:**
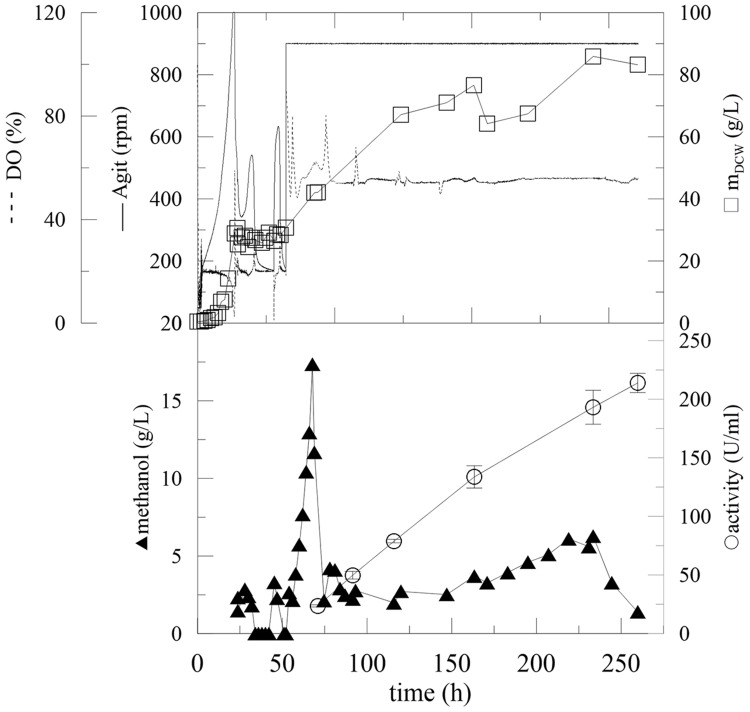
**Fermentation of *P. pastoris* expressing α-L-rhamnosidase with fed-batch methanol feeding according to actual level of dissolved oxygen.** Conditions: 1.5 L BSM, 30°C, pH 5 (ammonia solution), DO = 20% by agitation cascade 50–1,000 rpm, 5% inoculum.

It was obvious that the metabolic activity and biomass production of *P. pastoris* and the expression of the enzyme were limited by the residual concentration of methanol in the media. Furthermore, the metabolic activity and complete utilization of methanol by the biomass resulted in increased oxygen, as detected by the DO probe (**Figure 5**). Also, due to the higher methanol concentration the biomass concentration remained constant. Therefore, methanol feeding was linked to oxygen consumption in subsequent experiments. After glycerol utilization and the two methanol adaptation steps, agitation was adjusted at 900 rpm, providing constant oxygen saturation to *Pichia* and methanol feeding was linked to DO saturation. To simplify the experimental set up and operation of the fermentation process, a fully automated program was developed. The program was based on excess oxygen supply to the biomass and linked with methanol feeding, which was linked with a DO probe signal according to the following program: when the level of DO increased (methanol was consumed, the metabolic activity decreased) the methanol feeding rate increased automatically according to the following feeding program: if DO was <50%, methanol flow was set to 2 mL/h; DO = 50.01–52% methanol flow was 2.48 mL/h, DO = 52.01–54% methanol flow was 3.75 mL/h and DO = 54.01–56% methanol flow was 5.14 mL/h. To prevent the accumulation of methanol to critical toxic concentrations, the program was automatically switched off at DO saturations higher than 70%. This cascade program can be easily set up at any bioreactor with DO probe and pump which is operated with bioreactor software. Moreover, no on/off-line methanol monitoring and feeding is required with this method. At the beginning of the methanol-feeding program, when the agitation was set to 900 rpm and when the DO increased exponentially, the initial addition of methanol (17.4 g/L in the 55th hour) was essential to start the automated process (**Figure 6**). Almost immediately, the automated feeding program started. Compared with previous fermentations, the activity in the 200^th^ hour was 164.7 U/mL, which was 28 U/mL higher than the best experiment achieved so far (**Figure 6**; **Table 3**, Batch G). Furthermore, the limited amount of methanol in the fermentation broth (measured off-line by HPLC) improved the biomass production in the induction phase.

#### Up Scale of Fermentation

**FIGURE 7:**
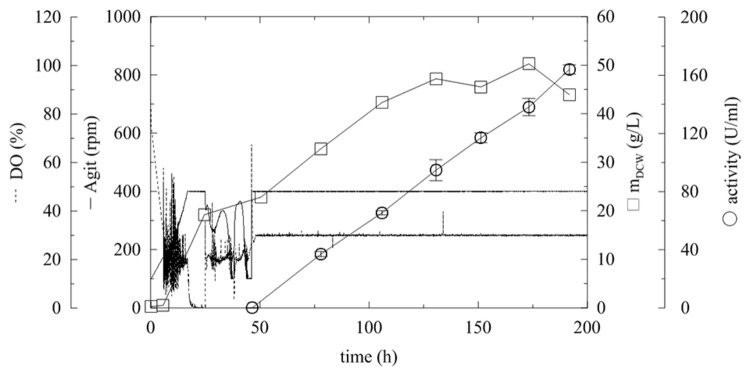
**Upscale fermentation of *P. pastoris* expressing α-L-rhamnosidase with fed-batch methanol feeding according to actual level of dissolved oxygen.** Conditions: 10 L BSM, 30°C, pH 5 (ammonia solution), DO = 20% by agitation cascade 100–400 rpm, 5% inoculum.

**FIGURE 8:**
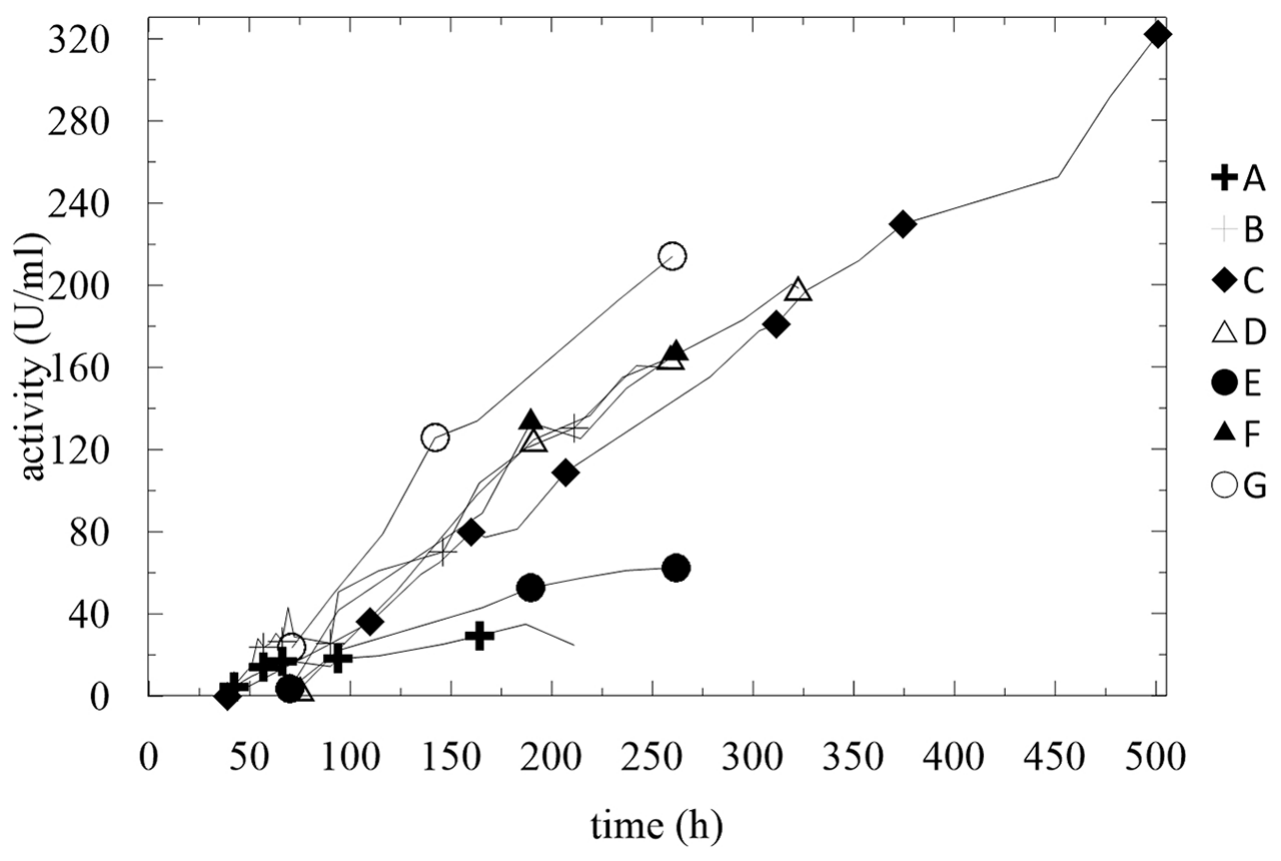
**Summary of activities of produced enzymes.** Conditions: 1.5 L BSM, 30°C, pH 5.0 (ammonia solution), DO = 20% by agitation cascade at 50–1,000 rpm.

To demonstrate the production of this industrially interesting and important enzyme, an upscale of the fermentation process in the *in situ* autoclavable 19.5 L laboratory bioreactor was performed. The optimized automatic program of methanol feeding was applied to a 10 L fermentation process (**Figure 7**; **Table 3**, Batch H). The maximum agitation cascade was set to 400 rpm, and the lack of oxygen was balanced by overpressure of 0.5 bar and aeration of 1 vvm. After the glycerol consumption phase, two methanol pulses were performed, and the agitation was then fixed to 400 rpm and the methanol fed-batch was started. Methanol feeding was based on actual DO level as described above, and it was also supplemented by PTM_1_ solution. To drop the methanol at the beginning of the fed-batch (approximately 46th hour), the initial pulse of 30 g of methanol was performed. When the DO level decreased below 30%, the automated methanol-feeding program was set to: if the DO was >30%, the methanol flow was set to 69 mL/h, and if the DO was >40%, the feeding was stopped. In this way, the culture only received the required methanol amount, and as expected, the current (off-line measured) methanol concentration of 0 g/L was observed throughout the entire experiment. This feeding rate resulted in biomass growth and also in a linear increase of α-L-rhamnosidase activity (**Figure 7**), which is comparable to the small-scale fermentation G (**Figure 8**; **Table 3**, Batch G). The total amount of produced proteins was 2 mg/mL with the specific activity of 82 U/mg in the 196th hour of fermentation and specific productivity of 13.34 mg/L/h, which is comparable with productivities achieved in Mut^S^ *P. pastoris* strains (D’Anjou and Daugulis, 2001; Xie et al., 2003). Compared with α-L-rhamnosidases expressed in *E. coli* where specific activity 56 U/mg was achieved (Zhang et al., 2015), almost 1.5× higher specific activity was achieved in *P. pastoris*. Screening the wild strain producers, the maximum achieved activity with *p*-nitrophenyl-α-rhamnoside as a substrate it was 1.32 U/ml by *Fusarium sambucinum* and 3.9 U/mg by *Trichoderma longibrachiatum* (Scaroni et al., 2002). Another study described a wild strain producers of α-L-rhamnosidase, where after various purification steps the maximum specific activity of 33.9 U/mg (*p*NP-α-L-Rha as a substrate) was achieved (Yanai and Sato, 2000).

Thus, this program is perfectly suitable for automated fermentation regulation without any methanol off-line measurement throughout the entire fermentation process, which was necessary in other *P. pastoris* fermentations (Curvers et al., 2001; Khatri and Hoffmann, 2006).

The experimental findings of the fermentations in BSM medium are summarized in **Figure 8** and **Table 3**. Except for fermentation E and A, where the concentration of methanol exceeded 60 g/L, all other activities increased linearly with nearly the same trend, despite all the different conditions.

With almost all of the feeding strategies, the only parameter that significantly influenced the enzyme activity was high OD and a low concentration of methanol in the fermentation broth. We found that an inoculum of 5% v/v was a sufficient initial biomass load for the process, the optimum starting concentration of glycerol was 40 g/L, and methanol feeding may be operated at the continuous or the pulse mode but with a maximum final concentration of 3.5 g/L. Higher concentrations of methanol significantly inhibited the expression of the enzyme.

#### Long-term Enzyme Stability

Enzyme stability is a key factor for industrial applications (e.g., rutin derhamnosylation; Rebroš et al., 2013), debittering of fruit juices (Yadav et al., 2010), or vine aroma release (Yadav et al., 2010). Therefore, the fermentation broth (without biomass) from fermentation G was stored at 4°C for 18 months. The enzyme retained 95% of the original activity after 6 months and after 18 months the activity was still 73%. This clearly demonstrates that the enzyme is extremely stable and can be used even after several months of storage.

## Conclusion

Various methanol-feeding strategies were tested for the production of recombinant α-L-rhamnosidase by *P. pastoris* Mut^S^ (methanol utilization slow) strain. The optimal strategy was the application of automated methanol feeding connected to the on-line level of dissolved oxygen. This process and feeding strategy was successfully upscaled to a 10-L working volume, with no significant change in the production process. An easy handling fermentation protocol was developed, with no need of oxygen adjustment or on/off-line methanol monitoring during all the induction phase. Since oxygen probes are standard bioreactor components, this protocol simplifies the production of extracellularly produced enzymes using *P. pastoris* Mut^S^ strain. In this study the specific activity of α-L-rhamnosidase increased from 35 U/mg up to 82 U/mg in the up scaled fermentation, which is the highest specific activity of other recombinant and also wild producers described so far.

## Author Contributions

KM and MR performed the fermentation experiments and analyzed the data. MR designed the experiments. VK and LW provided the recombinant strain and optimized the analysis and enzyme activity assay. MR supervised the research. All authors read and approved the final manuscript.

## Conflict of Interest Statement

The authors declare that the research was conducted in the absence of any commercial or financial relationships that could be construed as a potential conflict of interest.
